# Genome-wide survey of the phosphofructokinase family in cassava and functional characterization in response to oxygen-deficient stress

**DOI:** 10.1186/s12870-021-03139-7

**Published:** 2021-08-16

**Authors:** Haiyan Wang, Pingjuan Zhao, Xu Shen, Zhiqiang Xia, Xincheng Zhou, Xin Chen, Cheng Lu, WenquanWang

**Affiliations:** 1 The Institute of Tropical Bioscience and Biotechnology (ITBB), Chinese Academy of Tropical Agricultural Sciences (CATAS), Haikou, 571101 P. R. China; 2 Key Laboratory of Biology and Genetic Resources of Tropical Crops, Ministry of Agriculture, Haikou, 571101 P. R. China; 3 College of Tropical Crops, Hainan University, Haikou, 570228 P. R. China

**Keywords:** Cassava, Phosphofructokinase, Oxygen deficiency, Expression profile

## Abstract

**Background:**

Glycolytic pathway is common in all plant organs, especially in oxygen-deficient tissues. Phosphofructokinase (PFK) is a rate-limiting enzyme in the glycolytic pathway and catalyses the phosphorylation of fructose-6-phosphate to fructose-1,6-bisphosphate. Cassava (*M. esculenta*) root is a huge storage organ with low amount of oxygen. However, less is known about the functions of PFK from *M. esculenta* (MePFK). We conducted a systematic analysis of *MePFK* genes to explore the function of the *MePFK* gene family under hypoxic stress.

**Results:**

We identified 13 *MePFK* genes and characterised their sequence structure. The phylogenetic tree divided the 13 genes into two groups: nine were *MePFK*s and four were pyrophosphate-fructose-6-phosphate phosphotransferase (*MePFP*s). We confirmed by green fluorescent protein fusion protein expression that MePFK03 and MePFPA1 were localised in the chloroplast and cytoplasm, respectively. The expression profiles of the 13 *MePFK*s detected by quantitative reverse transcription polymerase chain reaction revealed that *MePFK02*, *MePFK03*, *MePFPA1*, *MePFPB1* displayed higher expression in leaves, root and flower. The expression of *MePFK03*, *MePFPA1* and *MePFPB1* in tuber root increased gradually with plant growth. We confirmed that hypoxia occurred in the cassava root, and the concentration of oxygen was sharply decreasing from the outside to the inside root. The expression of *MePFK03*, *MePFPA1* and *MePFPB1* decreased with the decrease in the oxygen concentration in cassava root. Waterlogging stress treatment showed that the transcript level of PPi-dependent *MePFP* and *MeSuSy* were up-regulated remarkably and PPi-dependent glycolysis bypass was promoted.

**Conclusion:**

A systematic survey of phylogenetic relation, molecular characterisation, chromosomal and subcellular localisation and cis-element prediction of MePFKs were performed in cassava. The expression profiles of MePFKs in different development stages, organs and under waterlogging stress showed that MePFPA1 plays an important role during the growth and development of cassava. Combined with the transcriptional level of *MeSuSy*, we found that pyrophosphate (PPi)-dependent glycolysis bypass was promoted when cassava was under waterlogging stress. The results would provide insights for further studying the function of MePFKs under hypoxic stress.

**Supplementary Information:**

The online version contains supplementary material available at 10.1186/s12870-021-03139-7.

## Background

Hypoxia is common in plant tissues. Oxygen falls to very low level in metabolically active or large sink tissues. Low internal oxygen levels have been found in many plants, such as growing tuber [1], developing seeds [2], fruits [3], root [4] and phloem tissue [5]. Low oxygen concentration in the phloem would interfere with sugar retention [6]. The size of *Arabidopsis* seed shows a notable reduction when the external oxygen concentration drops below 15%, and seed production is remarkably inhibited when the oxygen concentration drops to 2% [7].

Glycolysis is an important metabolic pathway in all organisms. Glycolysis provides energy for cell metabolism and help cells adapt to abiotic stresses, such as hypoxia, cold and drought. In classical plant glycolytic pathway, phosphofructokinase (PFK) is the main rate-limiting enzyme and regulatory point. PFK catalyses the phosphorylation of D-fructose-6-phosphate (F-6-P) to D-fructose-1,6-bisphophate (F-1,6-BP). Plants have two forms of PFK based on phosphoryl donors, namely, the ATP-dependent PFK (EC 2.7.1.11) and the pyrophosphate (PPi)-dependent pyrophosphate-fructose-6-phosphate phosphotransferase (PFP, EC 1.7.1.90) [8]. PFK catalyses the irreversible phosphorylation of F-6-P to F-1,6-BP, and PFP catalyses the reversible phosphorylation of F-6-P to F-1,6-BP. PFK remains very complex in higher plants. Latzko and Kelly found the PFK isoenzymes in spinach as early as 1977 [9]. Since then, PFK from various plant sources have been studied. PFKs are found in plastids and cytosol in many plants, such as tomato fruit [10], *R. communis* seeds [11] and potato [12]. Winkler firstly purified PFK protein from spinach leaves and identified the existing PFK and PFP in the spinach genomic database [8]. PFK from growing potato contains four polypeptides with molecular weight between 46,300 and 53,300. PFK’s isolation during the purification process is unstable; thus, study on PFK biochemistry is relatively few. Plant PFP has been characterised in much detail in many plants, such as *S. tuberosum* [13], *R. communis* [14] and *S. officinarum* [15]. PFP is common in all plant tissues, and its enzyme activity is usually equal to or greater than that of PFK [16]. PFP consists of two different subunits, namely, alpha and beta, which form a heterotetramer [12]. In potato, PFP is composed of two polypeptides with molecular weight between 58,000 and 55,700.

PFP is a self-adaptive enzyme, and its activity and subunit composition depend on environmental, developmental, tissue-specific and species conditions. PFP has multiple functions, including glycolysis, gluconeogenesis, regulation of PPi concentration in sucrose metabolism and plant adaptability to environmental changes. In *Arabidopsis* plants with PFP RNA interference, PFP activity is reduced and the growth of plants is severely inhibited [17]. The result shows that AtPFP is involved in carbohydrate metabolism. The transcript levels of several *PFKs* and *PFPs* from *O. sativa* (*OsPFKs* and *OsPFPs*) roots are increased during anoxia [18]. This phenomenon shows that PFP may be involved in glycolysis metabolism to help plants adapt to poor environment, such as drought, cold or low oxygen stress. In antisense potato plants of *PFP*, *PFP* expression is decreased remarkably with more than 90% reduction in PFP activity in growing tuber and stored tubers, but no visible phenotype is observed [19]. The decrease in PFP expression resulted in the decreased concentrations of phosphoenolpyruvate and glycerate-3-phosphate. Fructose-6-phosphate,2-kinase was stimulated to increase fructose-2,6-bisphosphate concentration, and ATP-dependent PFK was stimulated to compensate the decrease in PFP protein. The activity is increased remarkably in germinated coleoptiles and the suspension cell of rice under hypoxic stress, but the activity of PFK does not change [20]. Although PFP has received much attention, the function of PFP has not been well elaborated.

Progress in sequencing technology has enabled the whole-genome sequencing of many plant species. *PFK* gene families have been characterised in some plants, such as *Arabidopsis* [21], spinach [8], *Saccharum* [22], rice [20] and Chinese white pear [23]. Eleven members of the *PFK* gene family are present in *A. thaliana*: four members belong to *AtPFP*, and seven belong to *AtPFK*. Fifteen *PFK* genes are present in rice; five belong to *OsPFP*, and 10 belong to *OsPFK*. Fourteen members are present in white pear (*Pyrus bretschneideri*) *PFK* family: 10 belong to *PbPFK*, and four belong to *PbPFP*. Overall, the PFK family has more than 10 members, and PFK is the centre in plant growth and development and has diverse functions.

Cassava (*M. esculenta* Crantz) is an important food crop apart from rice and maize in Africa and Asia. Cassava has tuber root with starch that provides dietary carbohydrate and is used to produce industrial starch and bioethanol [24]. Tuber root, which is a huge storage organ, is predicted to be hypoxic; thus, glycolysis will be prevalent in cassava tuber root, especially the pyrophosphate-dependent glycolysis pathway, which will save energy for the survival and metabolism of cassava. Although the PFK family has received much attention, the PFK family in cassava has not been reported yet. In this study, 13 *PFK* genes were identified from the cassava genome and divided into two groups on the basis of the conserved PFK domain. Their sequence structures and subcellular localisation were investigated. The transcript levels of the *PFK* family under normal condition and hypoxic stress were explored. Overall, this study identified several tissue-specific and hypoxic stress-responsive *PFK* genes, and these genes will be helpful for the study of hypoxic stress in cassava plant.

## Results

### Identification and phylogenetic analysis of *MePFK* genes in cassava

**Fig. 1:**
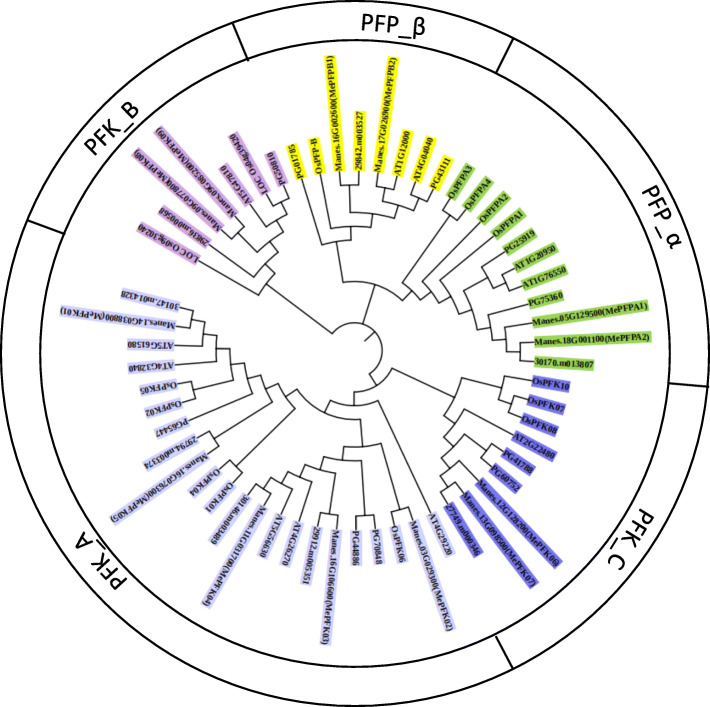
Phylogenetic relationship of *PFK* genes from cassava and four other species. The NJ tree was constructed with PFK proteins from cassava, *Arabidopsis*, potato, rice and castor bean using the Muscle programme of MEGA7 software with 1000 bootstraps. PFK protein was classified into two groups and five subgroups. The five subgroups are indicated with different background colours

Thirteen *MePFK* genes in the *M. esculenta* genome were identified using the online BLAST programme of JGI cassava genome data using the known *AtPFK* gene as reference. All of these genes contain conserved domain PF00365, which is the basic characteristic of the PFK family. A neighbour-joining (NJ) phylogenetic tree was drawn based on the multiple alignments of the MePFK amino acid sequences and other PFK sequences from *Arabidopsis*, rice, castorbeen, tomato and potato to investigate the evolutionary relationships between MePFK protein and other PFKs from other species (Fig. 1). Thirteen MePFK proteins were divided into two groups, namely, the MePFK and MePFP subfamilies. The MePFK subfamily was divided into three subgroups, namely, PFK-A, PFK-B and PFK-C. The MePFP subfamily was divided into two subgroups, namely, PFP-α and PFP-β. The 13 predicted MePFK proteins ranged from 318 amino acids (MePFPB2) to 617 amino acids (MePFKA1) (Table 1). The length of proteins varied distinctly; thus, different PFKs have different biological functions.

**Table 1:** Characteristics of the MePFK family

Gene Name	Accession NO.	DNA Length (bp)	Protein Length	Molecular Weight	PI	Grand average of hydropathicity	Num of Exon	Predicted localization
MePFK01	Manes.14G038800	5222	527	58,275.29	7.6	−0.211	14	Cytosolic
MePFK02	manes.03 g029300	4847	542	59,802.13	8.36	−0.241	14	Chloroplastic
MePFK03	Manes.16G106600	5713	557	61,105.55	7.15	−0.26	13	Chloroplastic
MePFK04	Manes.11G031700	3271	545	59,954.88	6.29	−0.278	13	Chloroplastic
MePFK05	Manes.16G076300	5566	481	52,794.94	6.41	−0.289	13	Cytosolic
MePFK06	Manes.12G128200	7202	530	58,186.72	7.89	−0.081	13	Chloroplastic
MePFK07	Manes.13G098500	10,556	536	58,917.32	7.62	−0.114	13	Chloroplastic
MePFK08	Manes.09G077800	3436	414	46,166.06	8.85	−0.129	4	Cytosolic
MePFK09	Manes.09G085200	2531	499	55,190.67	6.74	−0.221	2	Cytosolic
MePFPA1	Manes.05G129500	7660	617	67,407.42	6.42	−0.1	19	Cytosolic
MePFPA2	Manes.18G001100	6967	617	67,559.47	6.5	−0.128	19	Cytosolic
MePFPB1	Manes.16G002600	5872	566	61,675.9	6.15	−0.105	16	Cytosolic
MePFPB2	Manes.17G026900	3917	318	34,176.28	6.94	−0.005	8	Cytosolic

Molecular Weight:Molecular weight of the amino acid sequence; PI:Isoelectric point of the MePFKs; Grand average of hydropathicity:hydrophilic mean;Predicted localization: The sub-cellular localisation was predicted by analysing the sequences using the prediction programs the Cell-PLoc 2.0 package

### Exon–intron structure and motifs of *MePFK* were highly conserved

**Fig. 2:**
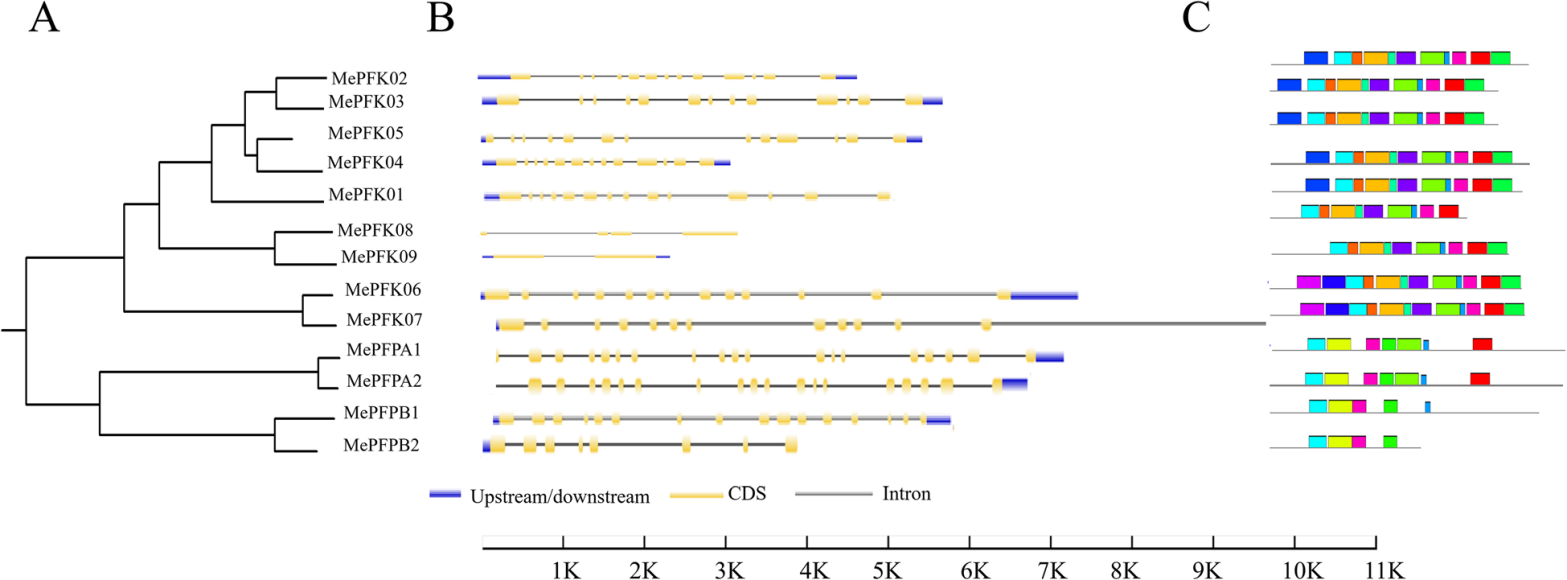
Phylogenetic tree, exon–intron structure and conserved motifs of the MePFK family. A, Phylogenetic tree of MePFKs in cassava. B, Exon–intron structure of *MePFK* genes. Yellow boxes represent CDS and grey lines represent introns. C, Conserved motifs in the MePFK protein. Each motif is represented by a different colour

We compared the exon–intron organisation in the coding sequences (CDSs) of *MePFK* genes to obtain a further insight into the structural diversity of the *PFK* genes. As shown in Fig. 2A, the members of *MePFK* with closely genetic relationship showed similar exon–intron structure. Most of the *MePFK* genes in cassava have more than 10 exons, except for *MePFK08*, *MeFPK09* and *MePFPB2*. *MePFK08* and *MePFK09* belong to PFK-B and only contain four and two exons, respectively. *MePFK08* and *MePFK09* have longer introns and exons than the other members. This special structure may be endowed with a special function. A total of 13–14 exons were found in the PFK-A and PFK-C subgroups. In the PFP subfamily, 19 exons were observed in *MePFPA1* and *MePFPA2*, 16 exons in *MePFPB1* and 8 exons in *MePFPB2*. The length of *MePFPB1* transcript was 2209 bp, and that of *MePFPB2* transcript was 2209 bp. By comparing the nucleotide sequences of *MePFPB1* and *MePFPB2*, it was found that they had high homology (89%, Fig. S1). The homologous region is located at base 153 to base 1111 of 5′ in *MePFPB1*. We inferred that *MePFPB2* is a mutation of *MePFPB1*, which stops transcription in advance.

We also predicted the conserved domains of all cassava MePFK protein sequences. Fifteen different motifs were identified in the *MePFK* gene families (Figs. S2 and B). Motif 2 was the common domain in all MePFKs. Motifs 10 and 11 were the exclusive domains of the PFP subfamily, and motif 14 was the exclusive domain of MePFK06 and MePFK07. Motifs 1, 3, 4, 5, 8, 9, 12 and 13 were the common domain of the PFK subfamily, and motifs 4, 5, 8, 9 and 12 were the exclusive domains of the PFK subfamily, which exists in series. All of which reflected that the PFK and PFP subfamilies have similar functions, and each has its own division of labour.

### Chromosomal and subcellular localisation of PFK in cassava

**Fig. 3:**
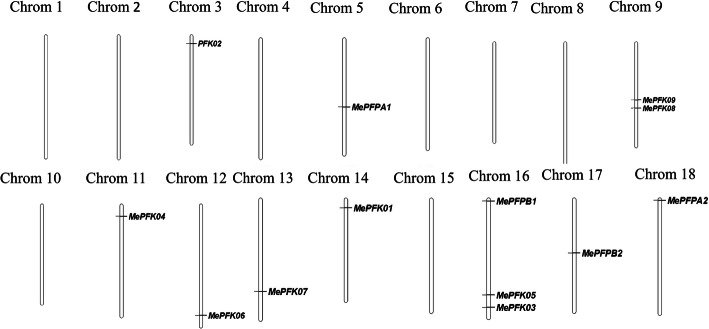
Distribution of *MePFK* genes in cassava chromosomes

The genomic distribution of *PFK* genes on the chromosomes of cassava was identified. A total of 13 *MePFK* genes were distributed throughout 10 of 18 chromosomes. Most of the 10 chromosomes contained one *PFK* gene, with chromosomes 9 and 16 containing two and three genes, respectively (Fig. 3).

**Fig. 4:**
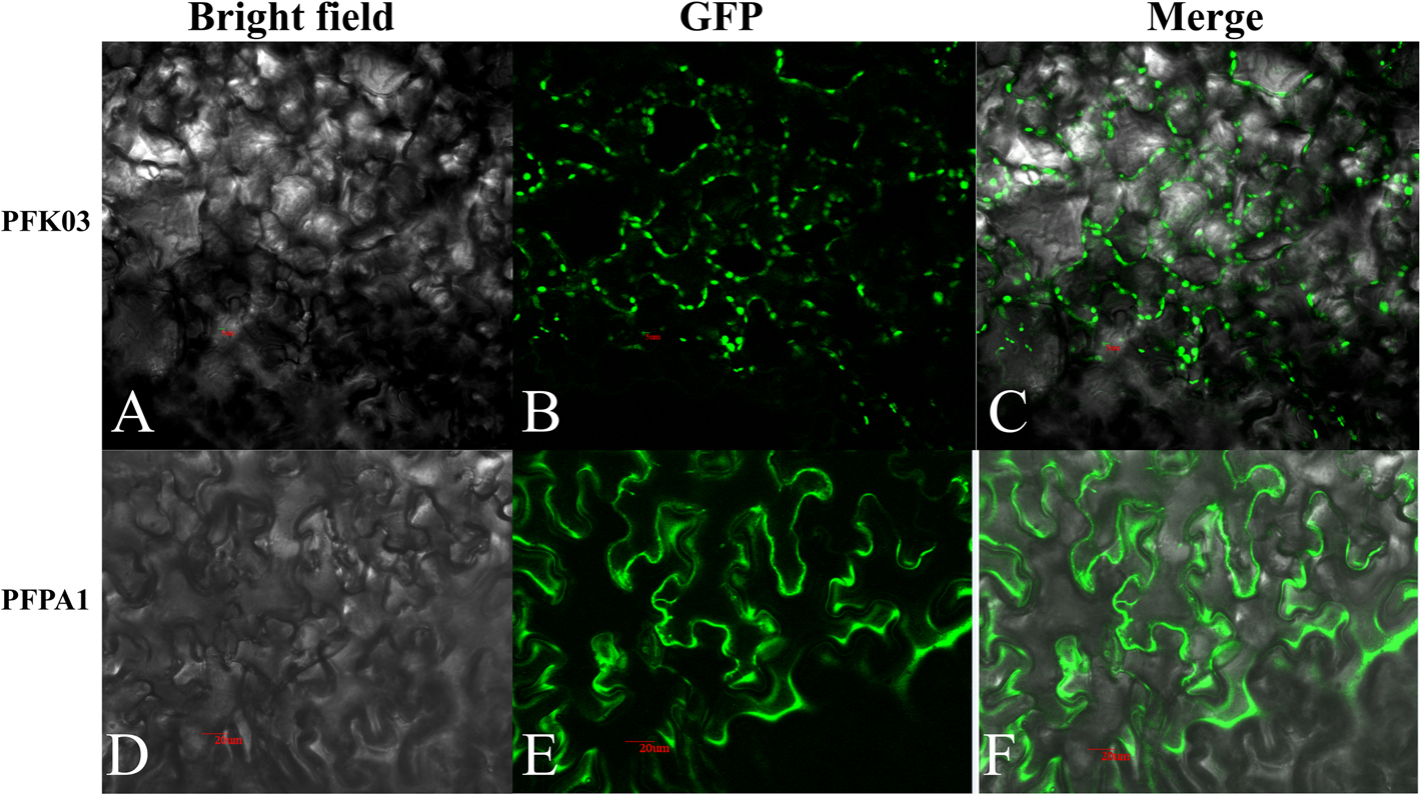
Subcellular localisation of 35S::MePFK03::GFP and 35S::MePFPA1::GFP fusion proteins in the lower epidermal cells of tobacco leaves. A, Bright-field image of the 35S::MePFK03::GFP fusion protein. B, GFP fluorescence of the 35S::MePFK03::GFP fusion protein. C, Merged A and B. D, Bright-field image of the 35S::MePFPA1-GFP fusion protein. E, GFP fluorescence of the 35S::MePFPA1::GFP fusion protein. F, Merged D and E

According to the online subcellular localisation software, five of the seven MePFKs were predicted to be localised at the chloroplast, two were predicted to be localised at the cytoplasm, and four PFPs were predicted to be localised at the cytoplasm (Table 1). Two PFKs (MePFK03 and MePFPA1), which belong to different groups and predicted to be located in the chloroplast and cytoplasm, respectively, were selected to construct MePFK03-GFP and MePFPA1-GFP fusion proteins to confirm the aforementioned result. Fluorescent signal results showed that MePFK03 was localised in the chloroplast and MePFPB1 was localised in the cytoplasm (Fig. 4). This finding is consistent with the predicted results.

### Cis-element prediction of cassava *MePFK* gene promoters

The cis-element within these promoters were identified using the online software new PLACE to better understand the functions of the *MePFK* genes. In addition to some common cis-elements, we found some special elements in *MePFKs* (Table 2). Amongst these elements, 17 elements were very typical, amongst which six belonged to organ-specific expression, seven were hormone-related, and the remainder were environment related. As for the organ-specific cis-elements, all the 13 PFK promoters contained many mesophyll DE expression modules; the maximum was 26, the least was 2, and most of them had more than 15. OSEROOTNODULE, which is a motif of specific activated elements in root modules, was widely distributed in 12 promoters; the number was up to 74 in *MePFK07* promoter but 0 in *MePFK08* promoter. Many ROOTMOTIFTAPOX (root-specific expression element) were distributed widely in 12 promoters but was 0 in the *MePFK06* promoter. Thus, most of were expressed in cassava root. POLLEN1LELAT52 (pollen-specific activation) was also widely distributed in 12 promoters but 0 in *MePFPB2*. A total of 28 RY-ELEMENT (specific in seed storage protein genes) were found in *MePFK08* promoter but few or none in the other promoters. Therefore, *MePFK08* had protein-related function in cassava. The cis-element prediction of these promoters indicated that MePFK was distributed in leaf, root and flower. Thus, the functions of the PFK lie in the leaf, root and flower. LTRE1HVBLT49 (low-temperature-responsive element) was distributed in 7 of 13 promoters. ANAERO1CONSENSUS/ANAERO3CONSENSUS (anaerobic-responsive element) was widely distributed in all 13 promoters, which reflected that MePFK was involved in hypoxic stress.

**Table 2:** Cis-element analysis of MePFK promoters

Motif name	Functoin of motif	MePFK01	MePFK02	MePFK03	MePFK04	MePFK05	MePFK06	MePFK07	MePFK08	MePFK09	MePFPA1	MePFPA2	MePFPB1	MePFPB2
CACTFTPPCA1	mesophyll DE expression module 1	20	21	17	21	16	2	15	20	26	17	7	25	21
GCN4 MOTIF	endosperm-specific expression	1	0	0	0	0	0	0	0	0	0	0	0	0
OSE2ROOTNODULE	motifs of organ-specific elements DE (OSE) characteristic of the promoters activated in infected cells DE of root nodules	6	6	8	10	9	5	3	0	5	3	1	10	8
ROOTMOTIFTAPOX1	root-specific expression	25	35	23	33	33	0	74	12	40	48	10	11	8
RY-ELEMENT	seed-storage DE protein genes in legume such as soybean	6	2		2				28				3	
POLLEN1LELAT52	pollen specific activation	16	13	24	14	15	3	8	8	19	12	4	6	0
ARR1AT	response DE regulator; N = G/A/C/T; AGATT is found in the promoter of rice DE non-symbiotic haemoglobin-2 (NSHB) gene (Ross et al., 2004);	33	20	20	20	24	5	11	7	27	19	5	22	38
GARE	GA-responsive element	0	1	0	0	0	2	0	0	3	3	0	0	
ABREZMRAB28/ABRERATERD1	ABA and water-stress responses	3	7	0	1	7	0	1	3	1	2	0	5	4
CATATGGMSAUR	Involved in auxin DE responsiveness	2	0	0	2	0	0	0	2	2	0	0	0	4
ARFAT	auxin response factor	1	1	1	1	1								
ERE	ethylene-induced DE activation of the U3 promoter region	1	1	3	1	2	0	0	0	1	1	1	0	
WRKY710S	a transcriptional repressor of the DE gibberellin signaling pathway	5	8	8	12	2	5	3	4	6	8	0	14	6
MYB related	responsive to DE water stress in Arabidopsis	9	19	21	7	15	12	10	16	16	11	2	30	16
LTRE1HVBLT49	low-temperature-responsive element	0	3	1	0	0	3	0	8	0	1	0	2	3
ANAERO1CONSENSUS/ANAER03CONSENSUS	anaerobic DE genes involved in the fermentative pathway	2	3	3	1	3	1	2	2	3	2	2	1	3
HSE	heat shock	0	0	2	0	0	2	0	0	0	0	0	0	
DOFCOREZM	binding of Dof proteins	31	22	38	35	26	8	9	31	18	26	5	28	28

Number of every selected cis-element in the MePFKs gene promoters. The yellow part indicated Tissue specific-relative elements; the orange part indicated hormone-relative elements; and the blue part indicated stress-relative elements

### Preliminary investigation on the oxygen concentration in cassava root

**Fig. 5:**
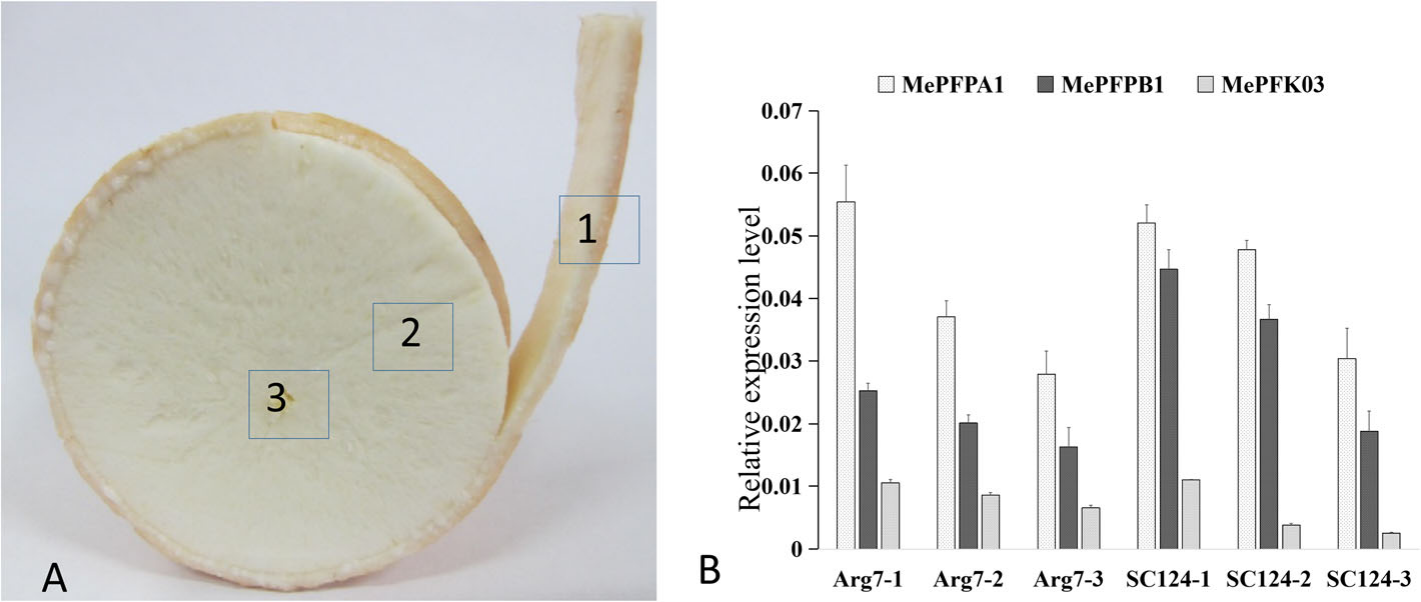
Schematic diagram of the different depths of cassava root and qRT-PCR analysis of three *MePFK* genes from the different depths of cassava root. 1 is under the periderm (sclerenchyma, parenchyma and phloem), 2 refers to the storage parenchyma, 3 refers to the tuber centre (xylem vessels and fibres)

According to existing literature [1–5], the active metabolism tissues of plant storage organs are often in the state of low oxygen. Therefore, cassava tuber root, which is a large storage organ, is supposed to be in low oxygen state. According to the transverse section of the root tuber, the middle segment of the tuber root in 1-year-old roots of different cassava varieties (Arg7 and SC124) was divided into three regions (Fig. 5A). Region 1 refers to the periderm and includes the sclerenchyma, parenchyma and phloem; region 2 refers to the parenchyma; and region 3 refers to the tuber centre and includes xylem vessels and fibres. The oxygen concentrations of regions 1, 2 and 3 were measured using an O_2_ microelectrode (Presens Company, Germany). The results are shown in Table 3. For Arg 7, the oxygen concentration under the periderm was 9–11.2%, and the concentration decreased to 2.58–3.96% in the centre. For SC124, the oxygen concentration under the periderm was 6.95–8.66%, and the concentration decreased to 3.7–5.2% in the centre, which showed a typical oxygen level profile throughout a growing tuber root. Overall, the oxygen concentration in root tuber decreased sharply from the outside to the inside. Compared with the oxygen concentration in the air (21%), hypoxia occurred in the inner cassava root.

**Table 3:** Internal oxygen concentration determination in different depths of cassava root

Individual plant	Diameter (cm)	Air oxygen concentration(%)	Oxygen concentration in region 1 (%)	Oxygen concentration in region 2 (%)	Oxygen concentration in region 3 (%)
Arg7–1	5.5	22.2	9^a^	5.28^b^	2.58^c^
Arg7–2	4.8	22.2	11.2 ^a^	7.28 ^b^	3.96 ^c^
Arg7–3	5.9	22.2	10.42 ^a^	8.35 ^b^	3.96 ^c^
SC124–1	6.4	22.2	6.95 ^a^	5.46 ^b^	3.7 ^c^
SC124–2	6.6	22.2	7.86 ^a^	6.31 ^b^	5 ^c^
SC124–3	9.3	22.2	8.66 ^a^	6.27 ^b^	5.2 ^c^

The middle segment of the tuber root was divided into three regions: region 1 refers to the periderm, which includes the sclerenchyma, parenchyma and phloem; region 2 refers to the storage parenchyma; and region 3 refers to the tuber centre, which includes xylem vessels and fibres. Values with different letters within one dividual are significantly different at *p* < 0.05

### Expression profiles of *MePFK* in cassava

**Fig. 6:**
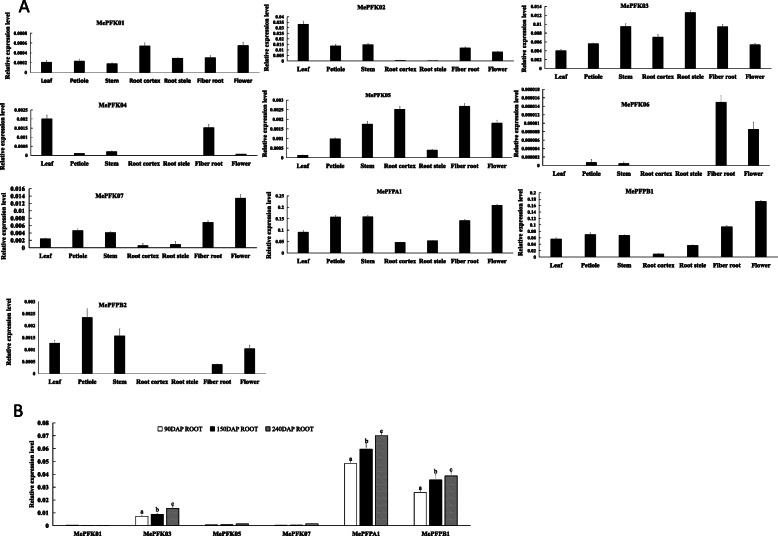
Expression analysis of 13 *MePFK* genes from different organs and from storage roots at three developmental stages. The x-axes indicate the different tissues and storage root of development stages of cassava. The y axes represent the expression fold relative to that of the internal reference gene. Error bars indicate the standard deviation calculated from three biological replicates. Different letters denote a significant difference at *p* < 0.05, which determined by the Duncan’s multiple range tests

The expression level of *MePFK* genes from different tissues, including leaves, petiole, stem, tuber root cortex, tuber root stele, fibre root and flower, were identified in SC124 to find some clues on the functions of PFK during the growth and development of cassava. As shown in Fig. 6A, *MePFPA1* and *MePFPB1* showed higher expression in all tissues than other genes. *MePFPA1* and *MePFPB1* were highly expressed in flower, leaf and fibrous root, which are metabolically active tissues. These genes also showed moderate expression in tuber root cortex and stele. *MePFKs* showed lower expression in all cassava tissues than *MePFPs*. Amongst *MePFKs*, *MePFK02* and *MePFK03* showed visible expression. The expression levels of *PFK* genes at three development periods of tuber root (90, 150 and 240 days after planting [DAP]) were also studied. As shown in Fig. 6B, *MePFK02*, *MePFK04*, *MePFK06*, *MePFK08*, *MePFK09*, *MePFPA2* and *MePFPB2* were almost not expressed in the root block. Thus, we only showed the higher expressed members of *MePFKs*. The expression levels of *MePFPA1*, *MePFPB1* and *MePFK03* increased gradually with the development of root tubers. This finding reflects that the functions of the three members were related to the development of tuber root.

*MePFPA1*, *MePFPB1* and *MePFK03* had relatively high expression in cassava root. Thus, they were selected to identify the expression pattern in cassava roots from different depths (Fig. 5B). The results showed that *MePFPA1* had the highest expression, *MePFPB1* had lower expression, and *MePFK03* had the lowest expression. A typical *MePFK* expression profile was shown through a growing tuber. The expression of *MePFK* was highest under the periderm and lowest in the centre. The expression levels of *MePFKs* were positively correlated with the change in oxygen concentration. This result indicates that the decrease in oxygen concentration affected the transcription of *MePFKs*.

### Transcriptional analysis of the key genes of glycolysis in cassava variety SC124 under waterlogging stress

**Fig. 7:**
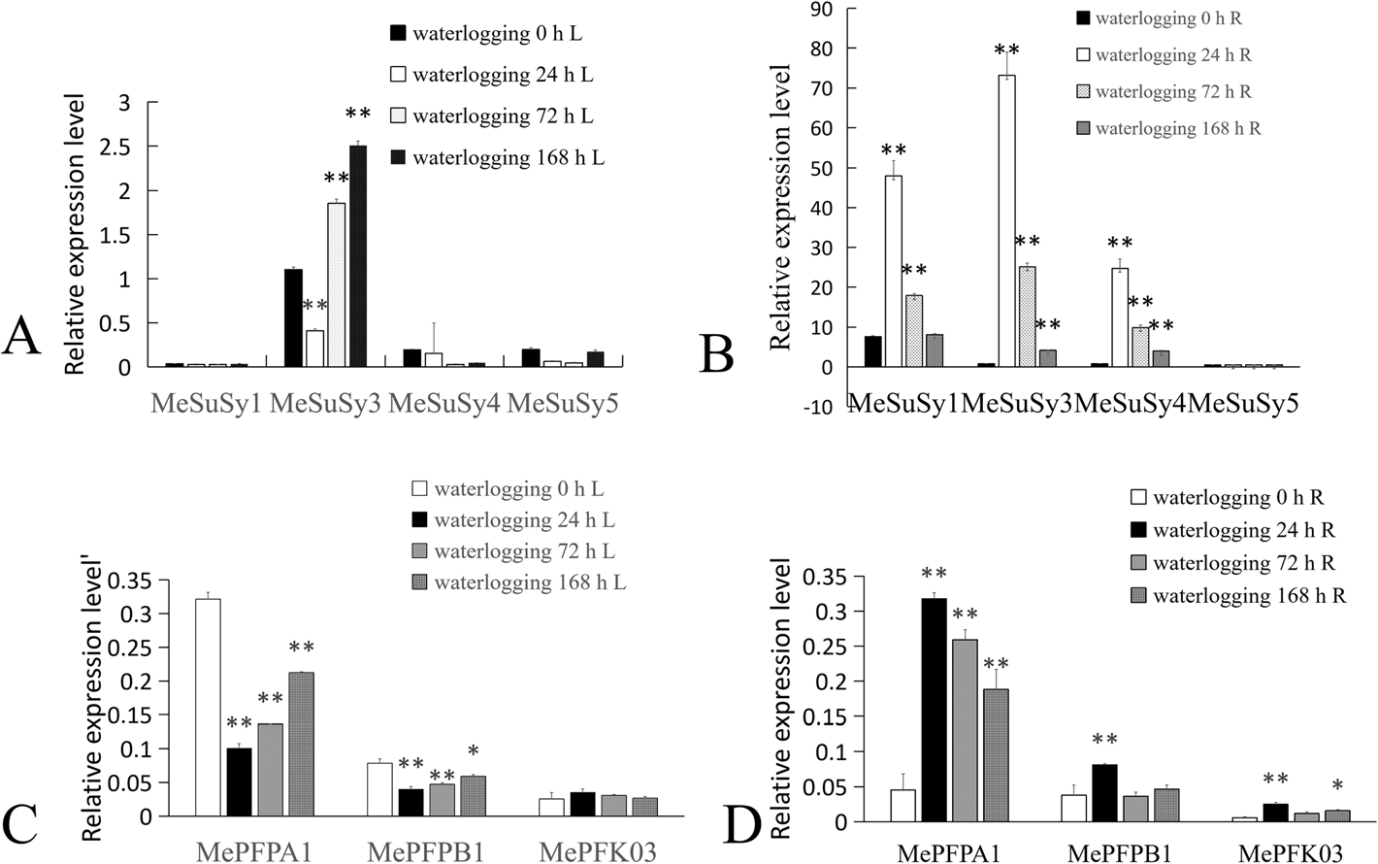
Expression profiles of *MeSuSy* and *MePFK* genes in the roots and leaves of cassava under different waterlogging times. A, Relative expression level of *MeSuSy* genes in leaves; B, Relative expression level of *MeSuSy* genes in roots; C, Relative expression level of *MePFK* genes in leaves; D, Relative expression profiles of *MePFK* genes in roots. The y axes indicate the expression fold relative to that of the internal reference gene. Error bars indicate the standard deviation calculated from three independent experiments. One and two asterisks (* and **)correspond to significant differences at *p <* 0.05 and *p* < 0.01, respectively

Considering the hypoxic stress in cassava tuber root, we designed an extreme anoxic environment. The roots of 5-month-old potted cassava plant (SC124) were waterlogged for 0, 24, 72 and 168 h, and the leaves and root tubers were used for the expression analysis of the key genes of glycolysis. We investigated the expression level of the *MeSuSy* family. The *MeSuSy* family has seven members, and the expression of *MeSuSy2*, *MeSuSy5*, *MeSuSy6* and *MeSuSy7* is very low [25]. In this study, the expression profile in Fig. 7A indicates that the expression of Me*SuSy3,* Me*SuSy4* and Me*SuSy6* in roots was remarkably increased under waterlogging stress compared with normal control. Among all the *MeSuSy* genes, only *MeSuSy5* was highly expressed in leaves. The expression of *MeSuSy5* was down-regulated in cassava leaves under waterlogging.

The expression profile of *MePFKs* under waterlogging stress (Fig. 7B) shows that the expression of *MePFPA1* in waterlogged leaves was down-regulated markedly, but that in waterlogged roots was up-regulated considerably. We can speculate that *MePFPA1* acts more in waterlogged roots and may be the main force that deals with hypoxic stress. The expression of *MePFPB1* in waterlogged leaves was up-regulated at 24 h of waterlogging and down-regulated at 72 h of waterlogging. Therefore, *MePFPB1* may act well at the early stage of waterlogging stress. The expression of *MePFK03* was lower in leaves than in roots; the expression of *MePFK03* in roots under waterlogging stress was up-regulated considerably but was nearly unchanged in waterlogged leaves. MePFK03 was responsive to waterlogged roots.

## Discussion

### Phylogenetic analysis of the *MePFK* family in cassava and four other species

We identified 13 members of the MePFK family in the whole genome of cassava. We submitted all MePFKs with four other PFK families from *Arabidopsis*, castor bean, rice and potato for phylogenetic tree analysis. The results showed that all *PFK* genes could be classified into PFK and PFP subfamilies. PFKs could be divided into PFK_A, PFK_B and PFK_C. PFPs could be divided into PFP_α and PFP_β. Amongst the 13 *MePFK* genes, nine belong to PFKs (five PFK_A, two PFK_B and two PFK_C) and four belong to PFPs (two PFP_α and two PFP_β). PFP is a heterotetramer with two different α and β subunits. Similar to *Arabidopsis*, cassava has two genes that encode α subunits, which are the regulatory subunits of PFP, and two genes that encode β subunits, which are the catalytically active subunits of PFP. Similar results were found in *Saccharum* [22], white pear [23] and rice [20].

### Expression analysis of *MePFKs*

The expression analysis of 13 *MePFKs* showed that *MePFPA1* and *MePFPB1* were highly expressed in seven tissue parts, including leaf, stem, petiole, fibre root, root cortex, root stele and flower. Therefore, *MePFPs* play important roles in the whole growth and development of cassava. PFP is involved in plant glycolysis [26–28]. PFP provides energy for plant morphogenesis and biochemical reaction through the glycolysis pathway. The expression level of MePFK genes in cassava were lower than that of MePFP genes. Among 9 MePFK genes, *MePFK02* and *MePFK03* showed relatively high expression. The highest expression for *MePFK02* was in the functional leaf, and that for *MePFK03* was in root tuber. Therefore, MePFK02 may be involved in glycolysis in leaves and MePFK03 may be involved in glycolysis in the tuber root expansion stage.

### Hypoxia occurred in the cassava tuber root

Hypoxia is a common phenomenon in plant tissues. We monitored the oxygen concentration in the roots of two cassava varieties (SC124 and Arg7). The results showed that the oxygen concentration decreased from the outside to the inside until the centre at a drop of below 5%. Similar phenomena were found in potato [29], castor [30], banana [31] and carrot [32]. The mechanism that can make plants conduct a series of biochemical reactions under hypoxia without causing internal anoxia is still unclear.

### Waterlogging stress in cassava variety SC124

We set up an experiment under extreme oxygen stress. Roots of cassava SC124 were completely immersed in water for 0, 24, 72 and 168 h. Then, leaves and tuber roots were collected for the transcription level analysis of the key genes of glycolysis. The immediate consequence of waterlogging was that oxygen was blocked and glycolysis flow was promoted under submerged condition. The expression level of *MePFK03* in tuber root was up-regulated under waterlogging stress. This finding is similar to that for *OsPFK05,* which showed a moderate increase in stem and leaves upon anoxia [20]. *MePFK03* and *OsPFK05* belong to the PFK_A subgroup in the evolutionary tree (Fig. 1). This inducible expression of *PFK* genes was also found in *Arabidopsis* (At4g26270 and At4g32480) [21]. Therefore, we can speculate that the induction expression of PFKs is an important regulation for plant metabolism under oxygen deficiency. As for PFP, the expression of *MePFPA1* in cassava root was up-regulated substantially under waterlogging stress, but the transcripts of *MePFPB1* showed up-regulated only at 24 h waterlogging. Similar trends were also observed in rice [20]. *OsPFPA* was more induced than *OsPFPB* in anoxic rice seedlings. PFPAs encode the regulatory subunit, and PFPBs encode the catalytic subunit. The adjustment of PFP activity is important under low oxygen stress, and PFPA plays a key regulatory role. The expression levels of different PFK isoforms are distinct in anoxic conditions, and this condition contributes to the balance of glycolysis capacity to cope with low oxygen stress.

**Fig. 8:**
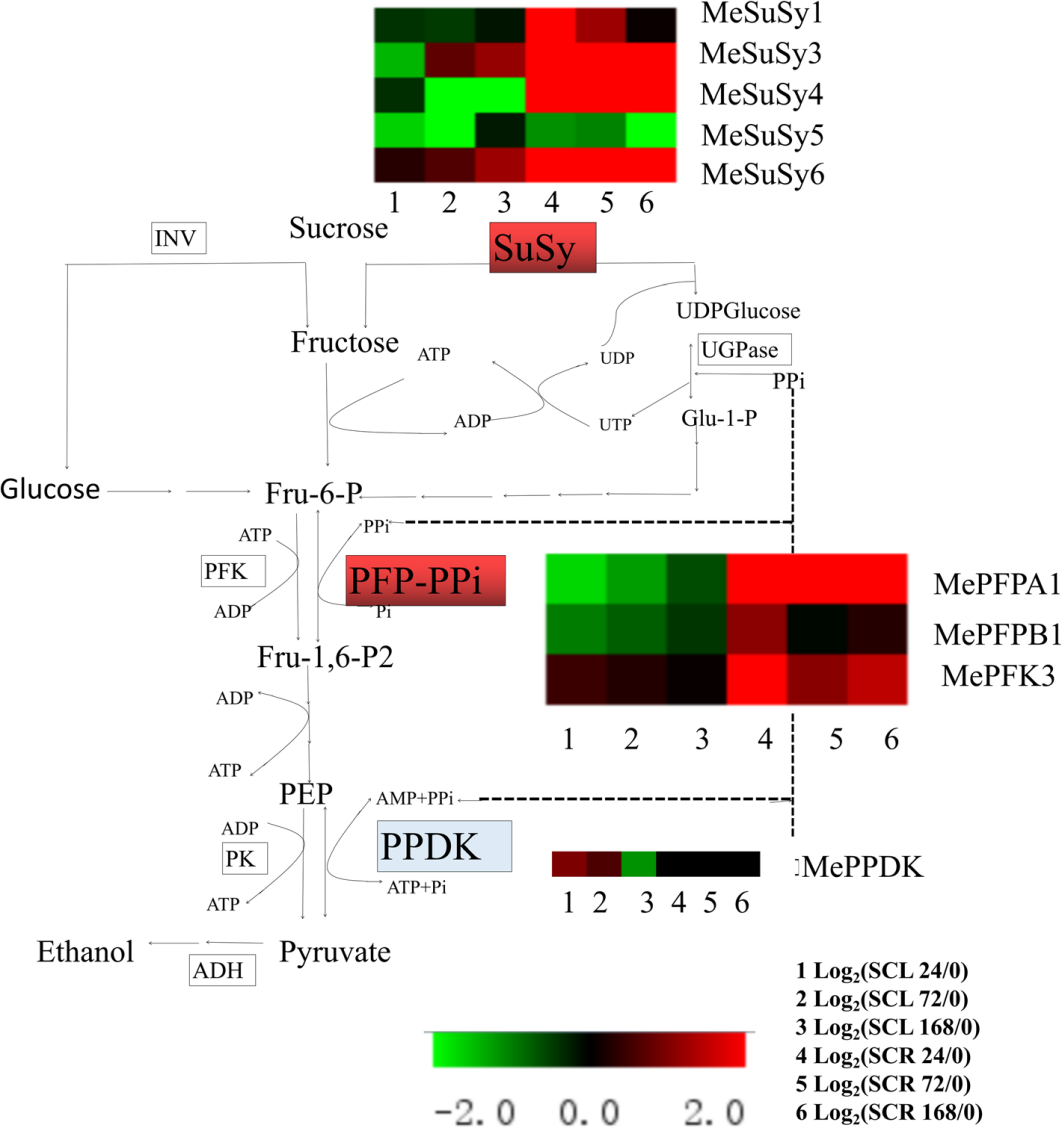
Expression of key genes involved in PPi-dependent glycolysis under waterlogging stress. Log_2_-based relative expression (24, 72 or 168 h/0 h) was used to create the heat map. Green represents down-regulation and red represents up-regulation in comparison with the values of untreated plants. The up-regulated enzymes are labelled with a red background

An oxygen-sensing system exists in plants under normal conditions, but hypoxia will lead to the decrease in adenylate energy level and respiration. Plants can optimise their metabolic pathways under hypoxia by saving ATP and improving the efficiency of oxygen utilisation. Two biochemical pathways from sucrose decomposition and glycolysis are present in plants, namely, the ATP-dependent conventional glycolysis and the PPi-dependent glycolysis. From the point of view of energy consumption, the glycolysis alternative dependent on PPi saves more energy. The three enzymes involved are sucrose synthase (SUSY), PFP and pyruvate orthophosphate dikinase (PPDK), which correspond to the enzymes of conventional glycolysis: invertase (INV), PFK and PK. When hypoxia stress begins, glycolysis will shift from the ATP-dependent pathway to the PPi-dependent alternative pathway [33, 34]. In this study, we investigated two genes of PPi-dependent glycolysis bypass. Firstly, sucrose decomposition can be catalysed by two enzymes, namely, the irreversible ATP-dependent INV or the reversible PPi-dependent SUSY. The second key step in glycolysis is the conversion of F-6-P to F-1,6-BP, which can also be catalysed by two enzymes, namely, the ATP-dependent PFK and PPi-dependent PFP. The transcription levels of *MeSuSy* and *MePFP* were up-regulated substantially in cassava root under waterlogging stress (Fig. 8). The third key step is the conversion of phosphoenolpyruvate to pyruvate, which can be catalysed by the ATP-dependent PK or the PPi-dependent PPDK. The expression of *MePPDK* in leaves was up-regulated after waterlogging treatment, but nearly no expression was observed in waterlogged root tubers [35]. The expression profiles of the above-mentioned enzymes indicate that the PPi-dependent glycolysis pathway was promoted in waterlogged root tubers, and this pathway may save energy and maintain the basic survival needs of plants.

In conclusion, 13 members were present in the MePFK family of cassava. These genes were distributed in 10 of 18 chromosomes. Nine belonged to MePFKs, and four belonged MePFPs. *MePFPs* showed higher expression in the organs of cassava than *MeFPKs*. The expression of *MePFK03*, *MePFPA1* and *MePFPB1* decreased gradually with the depth increase of cassava tuber. We also found that the expression of *MePFPA* and *MePFK03* increased remarkably in waterlogged roots. MePFPA1 plays an important role in cassava under waterlogging stress. This study will help suggest the candidate genes of how cassava adapts to hypoxic stress. The mechanism of PFP should be further determined by knocking out and overexpressing PFK in cassava root.

## Methods

### Plant materials and treatments

Materials for qRT-PCR. Cassava stalks (SC124 var.) were planted in Chengmai Experimental Field, Chinese Academy of Tropical Agricultural Sciences. The plants were grown for 180 days, and then different tissues from leaves, petiole, stem, root cortex, root stele, fibrous root and flower were collected and frozen in liquid nitrogen for RNA isolation and qRT-PCR. Tuber roots from different developmental stages at 90, 180 and 240 DAP were also collected and frozen in liquid nitrogen for RNA isolation and qRT-PCR.

Materials for waterlogging treatments. Cassava stalks (SC124 var.) were cut and placed in large pots. When the plants grew to 5 months old, the roots of cassava SC124 were completely immersed in water. The leaves and roots were collected at 0, 24, 72 and 168 h after waterlogging initiation and were frozen in liquid nitrogen for subsequent qRT-PCR.

### Sequence retrieval and phylogenetic tree construction

The whole protein sequence of the MePFK family was obtained from the cassava genome database (https://phytozome.jgi.doe.gov/pz/portal.html#!info?alias=Org_Mesculenta) [36]. We used the hidden Markov model profile of the PFK protein domains to search the database using the programme HMMER 3.0. The SMART online programme was used to assess the conserved domain of candidate PFKs (http://smart.embl-heidelberg.de/smart/set_mode.cgi? NORMAL = 1). Only proteins with PF00365 domain were regarded as PFK and reserved for further analysis. The multiple alignments of PFK proteins from five different species were made using the Muscle programme of MEGA7 software [37]. A bootstrap neighbour-joining (NJ) phylogenetic tree was established with 1000 bootstrap replicates. The phylogenetic tree of cassava was constructed to better exhibit PFK structure and conserved motifs.

### Sequence analyses and protein properties of MePFK and cis-element prediction of *MePFK* promoters

The isoelectric points and molecular weights of PFK proteins were examined using the ExPASy proteomics server (https://web.expasy.org/protparam/) [38]. *PFK* gene structures were identified by an online Gene Structure Display Server [39]. The MEME programme (http://meme-suite.org/tools/meme, Version 5.1.0) [40] was used to investigate the conserved motifs in PFK proteins. In the analysis, the maximum number of motifs was 15, and the optimum width of motifs was 6–50.

The promoter sequences (less than 2000 bp) of *MePFKs* were obtained from the cassava genomic sequence downloaded from the JGI database. These sequences were submitted to the new PLACE database (https://www.dna.affrc.go.jp/PLACE/?action=newplace) for the analysis of the cis-elements of *MePFK* promoters [41].

### Chromosomal and subcellular localization of MePFK protein in cassava

The chromosomal locations of *MePFK* were determined on the basis of the chromosomal information derived from the JGI database. The position of *MePFK* was physically mapped to a chromosome using the Mapchart software according to the relative location of *MePFK* on the chromosome [42]. The subcellular localisation of the MePFK protein was predicted online using the Cell-PLoc 2.0 package [43]. We selected two genes for subcellular localisation by GFP fusion protein expression to confirm the predicted results. The CDS fragments of *MePFK03* and *MePFPA1* without stop codon were amplified using cDNA from Ku50 leaves by gene-specific primers (Table S1) designed according to the CDS from the JGI database. The fragments were cloned into the binary vector pCAMBIA1300R (modified based on pCAMBIA1300). Positive clones were confirmed by DNA sequencing, and the expression vectors of 35S::MePFK-GFP and 35S::MePFPA1-GFP were generated. The expression vectors were transformed into *A. tumefaciens* EHA105 and then infiltrated into the leaves of *Nicotiana benthamiana* for transient expression. After 2 days, the infiltrated leaves were placed on a confocal laser scanning microscope to observe the GFP fluorescent signals.

### Oxygen concentration determination in cassava tubers

Oxygen concentration was analysed from two cassava varieties, namely, Arg7 and SC124. Three individual plants were collected from each variety, two tubers were sampled per plant, three regions were selected per tuber, and each region was tested 10 times. Internal oxygen concentration was measured 1–2 min later by inserting an O_2_ microelectrode (< 1 mm tip diameter; Presens, Germany) into the tuber root tissue. The measured air oxygen concentration was measured 22%. Tissues were sampled at the same region for the transcript analysis of *MePFKs.*

### Transcript analysis of *MePFKs i*n cassava plants

RNA extraction and cDNA synthesis. Total RNA was extracted from cassava tissues through the sodium dodecyl sulfate method [44], and purified by NucleoTrap® mRNA kit (Macherey Nagel, Genmany) according to the manufacturer’s instruction. First strand cDNAs were synthesized by SMARTScribe™ Reverse Transcriptase from 200 ng of total RNA using primers of CDS III and Oligo III according to product manual.

The expression levels of *MePFKs* from different cassava tissues and from roots at different developmental stages were investigated by qRT-PCR analysis. qRT-PCR was performed in the thermal cycler of a Rotor-Gene 6000 (Rcorbett, Australia) using a SYBR Premix Ex TaqTM fluorescence quantitative kit (TaKaRa, Japan). Tubulin-F: 5′GTGGAGGAACTGGTTCTGGA3′ and Tubulin-R: 5′TGCACTCATCTGCATTCTCC3′ were used as reference gene [45]. The gene-specific primers are shown in Table S1. The PCR programme proceeded as follows: 95 °C for 30 s, 40 cycles of 95 °C for 10 s, 59 °C for 20 s and 72 °C for 30 s. Each sample had three technical replicates. The relative expression level was determined by the 2^−Δ Δ Ct^ method [46].

### Statistical analysis

Three biological replicates were used for each measurement. The data are represented as mean ±standard deviation. Significant differences between different samples were tested with the software IBM SPSS statistics 19.0 [47].

## Supplementary Information

**Additional file 1: Fig. S1** Sequence alignment result of MePFPB1 and MePFPB2

**Additional file 2: Fig. S2** Schematic representation of the conserved domain in MePFK proteins.

**Additional file 3: Table S1** Primers used in qRT-PCR analysis and vector construction.

**Additional file 4: Table S2** Transcript sequences, protein sequences and promoter sequences of MePFKs.

**Additional file 5: Table S3** protein sequences of PFKs from Arabidopsis, rice, castorbeen, tomato and potato.

**Additional file 6: Table S4** Transcript sequences of six *MeSuSys*.

**Additional file 7: Table S5** The raw data of internal oxygen concentration determination in different depths of cassava root.

## Data Availability

The sequence information of phosphofructokinase genes of cassava, *Arabidopsis*, rice, castorbeen, tomato and potato is available in the public domain in the Phytozyme v12 database resource (https://phytozome.jgi.doe.gov/), and are presented in Supplementary file Table S2, Table S3, and the transcript sequence of MeSuSys are presented in Supplementary file Table S4. All images depicted in the fgures are our own. The raw data for oxygen concentration determination n different depths of cassava root are presented in Supplementary file Table S5. All other data are available from the corresponding author for noncommercial purposes.

## Abbreviations

PFK: Phosphofructokinase
PFP: Pyrophosphate-fructose-6-phosphate phosphotransferase
SuSy: Sucrose synthase
PPi: Pyrophosphate
PPDK: Pyruvate orthophosphate dikinase
GFP: Green fluorescent protein
INV: Invertase
F-6-P: Fructose-6-phosphate
F-1,6-BP: D-fructose 1,6-biphosphate
RT-PCR: Reverse transcription-polymerase chain reaction

## References

[1] Geigenberger P (2003). Response of plant metabolism to too little oxygen. Curr Opin Plant Biol.

[2] Gibon Y, Blaesing OE, Hannemann J, Carillo P, Höhne M, Hendriks JHM, Palacios N, Cross J, Selbig J, Stitt M (2004). A robot-based platform to measure multiple enzyme activities in *Arabidopsis* using a set of cycling assays: comparison of changes of enzyme activities and transcript levels during diurnal cycles and in prolonged darkness. Plant Cell.

[3] Ke D, Yahia E, Hess B, Zhou L, Kader AA (1995). Regulation of fermentative metabolism in avocado fruit under oxygen and carbon dioxide stresses. J Am Soc Hortic Sci.

[4] Thomsonc J, Atwell BJ, Greenway H (1989). Response of wheat seedlings to low O_2_ concentrations in nutrient solution: II. K +/Na+ SELECTIVITY OF ROOT TISSUES9. J Exp Bot.

[5] van Dongen JT, Schurr U, Pfister M, Geigenberger P (2003). Phloem metabolism and function have to cope with low internal oxygen. Plant Physiol.

[6] Gibbs J, Turner DW, Armstrong W, Sivasithamparam K, Greenway H (1998). Response to oxygen deficiency in primary maize roots. Ii. Development of oxygen deficiency in the stele has limited short-term impact on radial hydraulic conductivity. Funct Plant Biol.

[7] Porterfield DM, Kuang A, Smith PJ, Crispi ML, Musgrave ME (1999). Oxygen-depleted zones inside reproductive structures of Brassicaceae: implications for oxygen control of seed development. Can J Bot.

[8] Winkler C, Delvos B, Martin W, Henze K (2007). Purification, microsequencing and cloning of spinach atp-dependent phosphofructokinase link sequence and function for the plant enzyme. FEBS J.

[9] Kelly GJ, Latzko E (1977). Chloroplast phosphofructokinase: II. Partial purification, kinetic and regulatory properties. Plant Physiol.

[10] Isaac JE, Rhodes MJC (1987). The role of inorganic phosphate in the regulation of pfk activity in tomatoes. Phytochemistry..

[11] Podesta FE, Plaxton WC (1994). Regulation of cytosolic carbon metabolism in germinating *Ricinus communis* cotyledons. Planta..

[12] Teramoto M, Koshiishi C, Ashihara H (2000). Wound-induced respiration and pyrophosphate:fructose-6-phosphate phosphotransferase in potato tubers. Z Naturforsch C.

[13] Carlisle SM, Blakeley SD, Hemmingsem SM, Trevanion SJ, Hiyoshi T, Kruger NJ, Dennis DT (1990). Pyrophosphate-dependent phosphofructokinase. Conservation of protein sequence between the alpha- and beta- subunits and with the ATP-dependent phosphofructokinase. J Biol Chem.

[14] Todd JF, Blakeley SD, Dennis DT (1995). Structure of the genes encoding the α and β-subunits of castor pyrophosphate dependent phosphofructokinase. Gene..

[15] Suzuki J, Mutton MA, Ferro MIT, Lemos MVF, Pizauro FM, Mutton MJR, DiMauro SMZ (2003). Putative pyrophosphate phosphofructose 1-kinase genes identifified in sugar cane may be getting energy from pyrophosphate. Genet Mol Res: GMR.

[16] Hajirezaei M, Stitt M (1991). Contrasting roles for pyrophosphate:fructose-6-phosphate phosphotransferase during aging of tissue slices from potato tubers and carrot storage tissue. Plant Sci.

[17] Lim H, Cho MH, Jeon JS, Bhoo SH, Kwon YK, Hahn TR (2009). Altered expression of pyrophosphate: Fructose-6-phosphate 1-phosphotransferase affects the growth of transgenic Arabidopsis plants. Mol Cell.

[18] Mustroph A, Stock J, Hess N, Aldous S, Dreilich A, Grimm B (2013). Characterization of the phosphofructokinase gene family in Rice and its expression under oxygen deficiency stress. Front Plant Sci.

[19] Hajirezaei M, Sonnewald U, Viola R, Carlisle S, Stitt DM (1994). Transgenic potato plants with strongly decreased expression of pyrophosphate:fructose-6-phosphate phosphotransferase show no visible phenotype and only minor changes in metabolic fluxes in their tubers. Planta..

[20] Kato-Noguchi H (2002). The catalytic direction of pyrophosphate: fructose 6-phosphate 1-phosphotransferase in rice coleoptiles in anoxia. Physiol Plant.

[21] Mustroph A, Sonnewald U, Biemelt S (2007). Characterisation of the ATP-dependent phosphofructokinase gene family from Arabidopsis thaliana. FEBS Lett.

[22] Zhu L, Zhang J, Chen Y, Pan H, Ming R (2013). Identification and genes expression analysis of atp-dependent phosphofructokinase family members among three saccharum species. Funct Plant Biol.

[23] Lv H, Li J, Huang Y, Zhang M, Zhang S, Wu J (2019). Genome-wide identification, expression and functional analysis of the phosphofructokinase gene family in chinese white pear (pyrus bretschneideri). Gene..

[24] Zidenga T, Leyva-Guerrero E, Moon H (2012). Extending cassava root shelf life via reduction of reactive oxygen species production. Plant Physiol.

[25] Liu C (2018). Studies on the gene family of sucrose synthase and the transcriptional regulatory factors of sucrose synthase 1 gene in cassava [D]. Hainan University.

[26] Nakamura N, Suzuki Y, Suzuki H (1992). Pyrophosphate-dependent phosphofructokinase from pollen: properties and possible roles in sugar metabolism. Physiol Plant.

[27] Groenewald JH, Botha FC (2001). Manipulating sucrose metabolism with a single enzyme: pyrophosphate-dependent phosphofructokinase (PFP). Proc S Afr Sugar Technol Assoc.

[28] Funaguma T, Hibino Y, Fukumori S, Hara A (1987). Pyrophosphate- and ATP-dependent phosphofructokinases in pollen of Typha latifolia. Agric Biol Chem.

[29] Geigenberger P, Fernie AR, Gibon Y, Christ M, Stitt M (2000). Metabolic activity decreases as an adaptive response to low internal oxygen in growing potato tubers. Biol Chem.

[30] Gong Y, Mattheis JP (2003). Effects of low oxygen on active oxygen metabolism and internal browning in "braeburn" apple fruit. Acta Hortic.

[31] Banks NH (1983). Evaluation of methods for determining internal gases in banana fruit. J Exp Bot.

[32] Kato-Noguchi HK, Watada AE (1997). Effects of low-oxygen atmosphere on ethanolic fermentation in fresh-cut carrots. J Am Soc Hortic Sci.

[33] Bologa KL, Fernie AR, Leisse A, Loureiro ME, Geigenberger P (2003). A bypass of sucrose synthase leads to low internal oxygen and impaired metabolic performance in growing potato tubers. Plant Physiol.

[34] Geigenberger P (2003). Regulation of sucrose to starch conversion in growing potato tubers. J Exp Bot.

[35] Wang H, Liu C, Ma P, Li K, Wang W. Functional characterization of cytosolic pyruvate phosphate dikinase gene (MecyPPDK) and promoter (MecyPPDKP) of cassava in response to abiotic stress in transgenic tobacco. Crop Sci. 2018;58(5):2002–9.

[36] Bredeson JV, Lyons JB, Prochnik SE, Wu GA, Ha CM, Edsinger-Gonzales E, Grimwood J, Schmutz J, Rabbi IY, Egesi C, Nauluvula P, Lebot V, Ndunguru J, Mkamilo G, Bart RS, Setter TL, Gleadow RM, Kulakow P, Ferguson ME, Rounsley S, Rokhsar DS (2016). Sequencing wild and cultivated cassava and related species reveals extensive interspecific hybridization and genetic diversity. Nat Biotechnol.

[37] Kumar S, Stecher G, Tamura K (2016). Mega7: molecular evolutionary genetics analysis version 7.0 for bigger datasets. Mol Biol Evol.

[38] Gasteiger E, Hoogland C, Gattiker A, Duvaud S, Wilkins MR, Appel RD, et al. Protein Identification and Analysis Tools on the ExPASy Server. In: Walker JM, editor. The Proteomics Protocols Handbook: Humana Press; 2005. p. 571–607.

[39] Hu B, Jin J, Guo A, Zhang H (2015). Luo Jingchu, Gao Ge. GSDS 2.0: an upgraded gene feature visualization server. Bioinformatics.

[40] Bailey TL, Boden M, Buske FA, Frith M, Grant CE, Clementi L, Ren J, Li WW, Noble WS (2009). MEME SUITE: tools for motif discovery and searching. Nucleic Acids Res.

[41] Higo K, Ugawa Y, Iwamoto M, Korenaga T (1999). Plant cis-acting regulatory DNA elements (PLACE) database: 1999. Nucleic Acids Res.

[42] Voorrips RE (2002). MapChart: software for the graphical presentation of linkage maps and QTLs. J Hered.

[43] Chou KC, Shen HB (2010). Cell-PLoc 2.0: an improved package of web-servers for predicting subcellular localization of proteins in various organisms. Nat Sci.

[44] Tang C, Qi J, Li H, Zhang C, Wang Y (2007). A convenient and efficient protocol for isolating high-quality RNA from latex of Hevea brasiliensis (Para rubber tree). J Biochem Biophys Methods.

[45] Salcedo A, Zambrana C, Siritunga D. Comparative expression analysis of reference genes in field-grown cassava. Trop Plant Biol. 2014;7(2):1–12.

[46] Livak KJ, Schmittgen TD (2001). Analysis of relative gene expression data using real-time quantitative PCR and the 2− ΔΔCT method. Methods..

[47] Corporation IBM (2011). SPSS Statistics software. Release 19.0.

