# Supplementary figures and images for: Genome-wide survey of the phosphofructokinase family in cassava and functional characterization in response to oxygen-deficient stress
Source: BMC Plant Biol. 2021 Aug 16;21:376. doi: 10.1186/s12870-021-03139-7 (PMC8365977; doi:10.1186/s12870-021-03139-7)

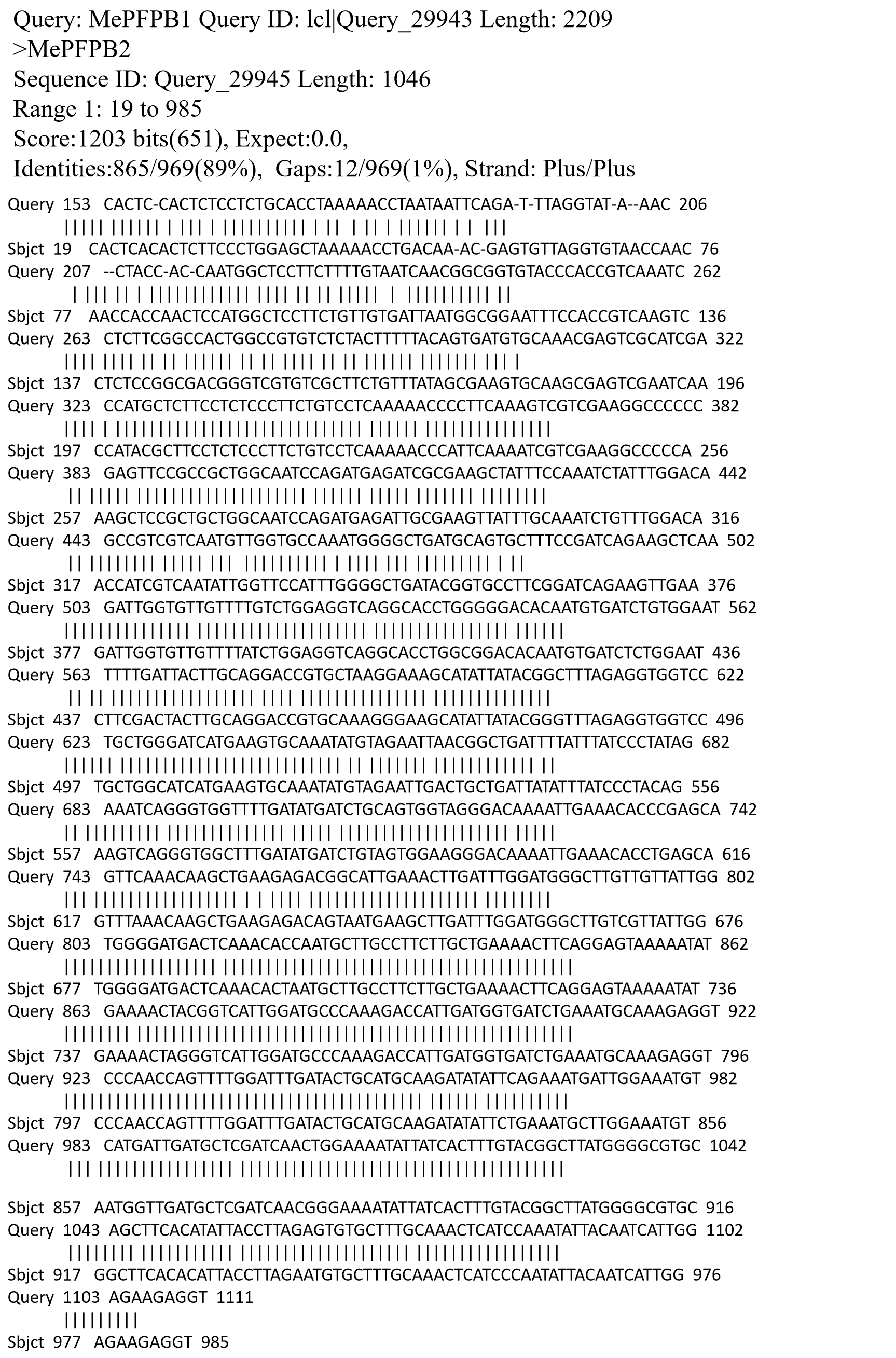

Supplement: Supplementary file 1 — Additional file 1: Fig. S1 Sequence alignment result of MePFPB1 and MePFPB2 [file 12870_2021_3139_MOESM1_ESM.tif]

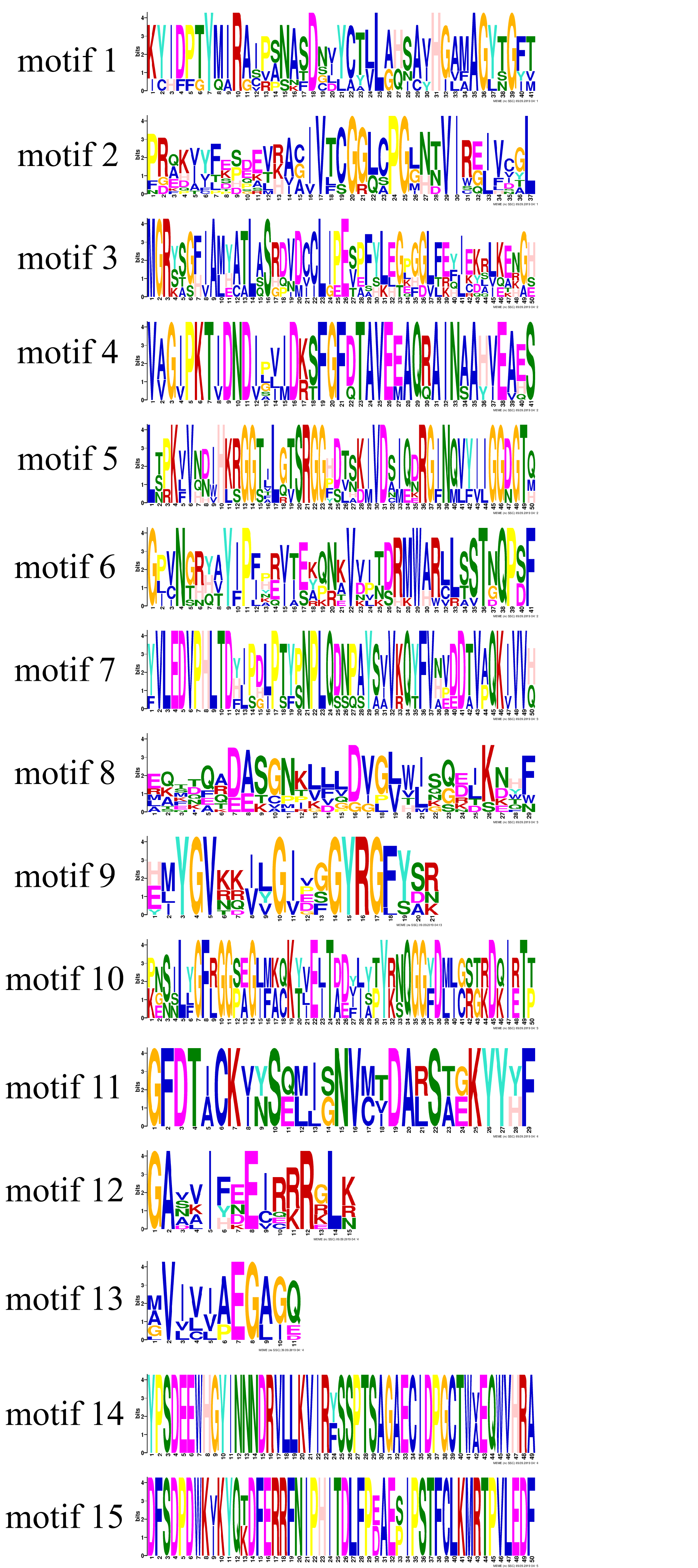

Supplement: Supplementary file 2 — Additional file 2: Fig. S2 Schematic representation of the conserved domain in MePFK proteins. [file 12870_2021_3139_MOESM2_ESM.tif]
